# Drug‐Free, Nonsurgical Reduction of Intraocular Pressure for Four Months after Suprachoroidal Injection of Hyaluronic Acid Hydrogel

**DOI:** 10.1002/advs.202001908

**Published:** 2020-12-07

**Authors:** J. Jeremy Chae, Jae Hwan Jung, Wei Zhu, Brandon G. Gerberich, Mohammad Reza Bahrani Fard, Hans E. Grossniklaus, C. Ross Ethier, Mark R. Prausnitz

**Affiliations:** ^1^ School of Chemical and Biomolecular Engineering Georgia Institute of Technology Atlanta GA 30332 USA; ^2^ Department of Pharmaceutical Engineering Dankook University Cheonan 16890 South Korea; ^3^ Wallace H. Coulter Department of Biomedical Engineering at Georgia Tech and Emory University Georgia Institute of Technology Atlanta GA 30332 USA; ^4^ Department of Pharmacology School of Pharmacy Qingdao University Qingdao 266021 China; ^5^ George W. Woodruff School of Mechanical Engineering Georgia Institute of Technology Atlanta GA 30332 USA; ^6^ Department of Ophthalmology Emory University School of Medicine Atlanta GA 30322 USA

**Keywords:** glaucoma, hyaluronic acid hydrogels, intraocular pressure, microneedle injections, suprachoroidal space

## Abstract

Glaucoma is the leading cause of irreversible blindness. Current treatments use drugs or surgery to reduce intraocular pressure (IOP). In this study, a drug‐free, nonsurgical method is developed that lowers IOP for 4 months without requiring daily patient adherence. The approach involves expanding the suprachoroidal space (SCS) of the eye with an in situ‐forming hydrogel injected using a microneedle. This study tests the hypothesis that SCS expansion increases the drainage of aqueous humor from the eye via the unconventional pathway, which thereby lowers IOP. SCS injection of a commercial hyaluronic acid (HA) hydrogel reduces the IOP of normotensive rabbits for more than 1 month and an optimized HA hydrogel formulation enables IOP reduction for 4 months. Safety assessment by clinical ophthalmic examinations indicate the treatment is well tolerated. Histopathology shows minor hemorrhage and fibrosis at the site of injection. Further analysis by ultrasound biomicroscopy demonstrates a strong correlation of IOP reduction with SCS expansion. Outflow facility measurements show no difference in pressure‐dependent outflow by the conventional pathway between treated and untreated eyes, supporting the hypothesis. In conclusion, SCS expansion with an in situ‐forming hydrogel can enable extended IOP reduction for treating ocular hypertension and glaucoma without drugs or surgery.

## Graphical abstract

The study reports a novel strategy for treating ocular hypertension and glaucoma with a drug‐free, nonsurgical procedure. The expansion of the suprachoroidal space with an in situ‐forming hyaluronic acid hydrogel administered using a microneedle reduced intraocular pressure of normotensive New Zealand white rabbits for approximately four months after a single injection without significant complications.

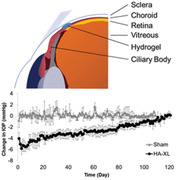

## 1 Introduction

An estimated 75 million people suffer from glaucoma, which is the world's leading cause of irreversible blindness.^[^ ^1^ ^]^ Vision loss in glaucoma involves the dysfunction and loss of retinal ganglion cell axons and is often associated with elevated intraocular pressure (IOP).^[^ ^2^ ^]^ In glaucoma, IOP elevation typically occurs due to impeded outflow of aqueous humor from the eye,^[^ ^3^ ^]^ which drains primarily through the trabecular meshwork located along the outer circumference of the anterior chamber.

Current glaucoma treatments fall into two main categories, both focused on controlling IOP. First is the use of medications (i.e., eye drops) to facilitate the outflow of aqueous humor and/or to decrease the production rate of aqueous humor.^[^ ^3^ ^]^ However, the need for repeated drug administration results in poor patient adherence that often translates into inadequate IOP control and, thus, disease progression.^[^ ^3^ ^]^ Further, patients often develop a tolerance for the treatment regimen over time. In this case, the second approach, namely surgical procedures, such as laser surgery, device implantation, or incisional surgery, are an alternative.^[^ ^4^ ^]^ However, surgeries are invasive, relatively expensive, and have reduced efficacy over time or upon repeated surgeries. Micro‐invasive glaucoma surgery may partially alleviate certain shortcomings of glaucoma surgeries by extending the efficacy period and by reducing discomfort, leading to a more rapid recovery.^[^ ^5^ ^]^ However, such *ab interno* surgical procedures can have serious side effects, such as damage to the corneal endothelium, are costly, and require surgical expertise often not available in developing countries.^[^ ^6^ ^]^ Hence, there remains a clinical need for a safe and effective option to lower IOP in glaucoma patients that is drug‐free, nonsurgical, and sustained, and ideally low‐cost and easy to administer. This approach should minimize the need for patient adherence, thereby helping to stop or delay the progression of vision loss.

In addition to the conventional pathway for aqueous humor clearance from the eye via the trabecular meshwork, the aqueous humor can also drain from the anterior chamber by uveoscleral (or unconventional) outflow.^[^ ^7^ ^]^ For example, the aqueous humor can pass through the extracellular matrix of the ciliary muscle into the suprachoroidal space (SCS). Increasing drainage via this pathway is an established strategy to reduce IOP, either pharmacologically by using prostaglandin analogues^[^ ^8^ ^]^ or surgically by placing a suprachoroidal drainage implant in the eye.^[^ ^9^ ^]^

**Figure 1:**
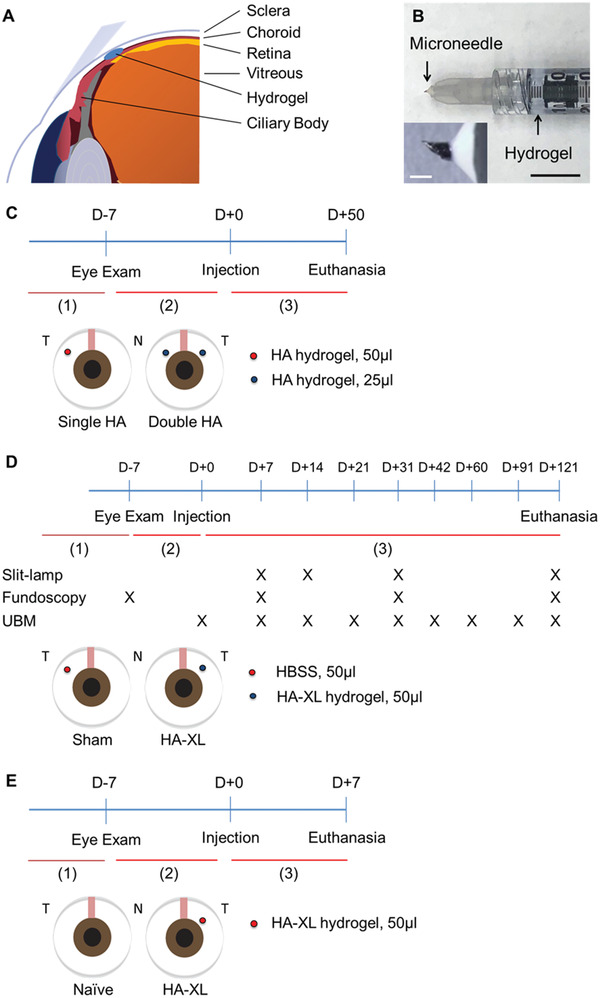
Overview of the suprachoroidal hydrogel injection protocol and study design. A) Schematic illustration of hydrogel injection (blue) into the suprachoroidal space located between the sclera and choroid adjacent to the ciliary body. B) Representative image of a microneedle used for injections (inset shows a magnified view). C) Design of the first proof‐of‐concept study using a commercial hyaluronic acid hydrogel (HA). D) Design of the second study to test whether the injection of an in situ‐forming HA hydrogel (HA‐XL) leads to extended intraocular pressure (IOP) reduction and to clinically evaluate the safety of the treatment. E) Design of the third study to explore the mechanism of IOP lowing by measuring outflow facility in eyes receiving HA‐XL compared to naïve control eyes. The studies were each divided into three periods, during which IOP was measured: 1) animal accommodation, 2) baseline IOP, and 3) post‐injection IOP. Abbreviations‐ D: Day; T: Temporal, N: Nasal, HBSS: Hanks Balanced Salt Solution; UBM: Ultrasound Biomicroscopy. Scale bar: 500 µm (White) and 1 cm (Black).

The SCS is a potential space between the choroid and sclera, forming part of the uveoscleral pathway.^[^ ^10^ ^]^ Recent studies have also shown the SCS to be a novel route for drug delivery to the chorioretinal layers of the eye^[^ ^11^ ^]^ With a hollow microneedle typically measuring ≤1 mm in length, drugs or agents can be simply and efficiently administered into the SCS without invasive procedures such as sclerotomy (**Figure** **1**B). After SCS injection, fluid can flow circumferentially within the SCS, bounded anteriorly by the ciliary body and posteriorly by the optic nerve. The distribution of material injected into the SCS can be controlled by formulation, such as viscous formulations that keep injected material near the site of injection and certain non‐Newtonian fluids that promote the distribution of injected material throughout the SCS.^[^ ^12^, ^13^ ^]^ Recent clinical trials have shown SCS injection to be well tolerated and carried out as a brief office procedure.^[^ ^14^ ^]^

Studies have shown that SCS injection can lead to alteration of IOP, typically involving a rapid transient increase upon injection, which dissipates within an hour. The magnitude of the IOP increase is similar to that seen upon routine intravitreal injection of an equal volume of fluid and is generally well‐tolerated.^[^ ^15^, ^16^, ^17^ ^]^ Over a longer time scale, IOP has been seen to drop below the baseline before returning to preinjection levels.^[^ ^18^, ^19^ ^]^ This drop lasts for a few days after injection of solutions with a viscosity close to that of water but was found to persist longer when highly viscous formulations were used in the rabbit.^[^ ^13^ ^]^

We hypothesize that this reduced IOP due to SCS injection is caused by the expansion of the SCS, which opens the uveoscleral outflow pathway and thereby facilitates drainage of aqueous humor from the anterior segment (Figure 1A). In this study, we designed formulations of hyaluronic acid (HA) hydrogel to maintain the expansion of the SCS for months after a single injection into the anterior SCS in order to provide long‐lasting IOP reduction. This method has potential as a novel, non‐surgical, and drug‐free treatment for treating ocular hypertension in glaucoma patients that does not rely on daily patient adherence.

## 2 Results

### 2.1 HA Formulation Was Designed to Expand SCS

This study tested the hypothesis that injection of hydrogel into the anterior SCS to expand the SCS of the rabbit eye can reduce IOP without the use of drugs or surgical procedures (Figure 1A). To test this hypothesis, we used a microneedle to inject an in situ‐gelling formulation of HA gel to expand the SCS and measured IOP over time (Figure 1B). Our motivation was the observation in earlier studies that hydrogels could effectively expand the SCS and lower IOP.^[^ ^13^ ^]^ By allowing gelation to occur within the SCS, the injection could be carried out using a low‐viscosity solution that subsequently formed a viscous gel and expanded within the SCS as it swelled with water. We selected HA to form the gel because it is naturally found in the eye as a component of vitreous humor and the extracellular matrix of the sclera, the choroid, the cornea, and other tissues.^[^ ^13^, ^20^ ^]^ It is also a biocompatible component of many formulations injected into the eye and other parts of the body.^[^ ^21^ ^]^

### 2.2 SCS Injection of Commercial HA Formulation Lowered IOP for 35 Days Compared to Baseline

**Figure 2:**
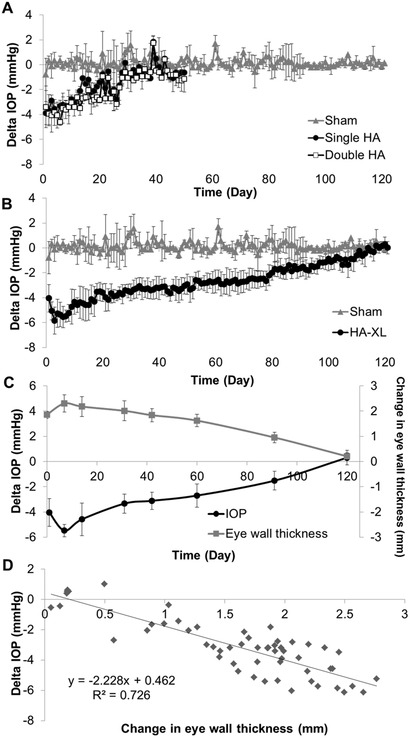
Effect of a suprachoroidal hydrogel injection on intraocular pressure (IOP) in the normotensive rabbit eye. A) A commercial hyaluronic acid (HA) hydrogel was injected into the suprachoroidal space (SCS) of the rabbit eye at one site (the superolateral quadrant, single HA group) or two sites (both the superolateral and superonasal quadrants, double HA group), or Hanks’ Balanced Salt Solution (Sham) was injected into the SCS of the rabbit eye at one side (the superolateral quadrant). Change in IOP compared to preinjection baseline values (Delta IOP) is shown. B) Delta IOP after an in situ‐forming HA hydrogel (HA‐XL) or Sham was injected into the SCS in the rabbit eye. C) Delta IOP after HA‐XL injection compared to the mean change in posterior eye wall thickness in the eye over time. Change in eye wall thickness is interpreted as the degree of SCS expansion D) A cross‐plot of change in posterior eye wall thickness versus Delta IOP (data from the graph in part C). Results are presented as mean ± standard deviation from seven eyes (HA‐XL group), two eyes per group (Sham, single HA, and double HA groups) or seven eyes per group (eye wall thickness). The same data for the sham‐injected eyes are shown in graphs (A) and (B).

As the first test of our hypothesis, we used a commercial HA hydrogel developed as a skin filler to treat wrinkles. Using a normotensive rabbit model, we observed that a single injection of 50 µL of this gel into the SCS led to an initial IOP reduction of ≈4 mmHg (**Figure** **2**A). Over time, IOP steadily approached its baseline value. Analysis by linear regression showed that the gel‐treated eyes had significantly lower IOP compared to their preinjected values for 35 days (*p* <0.01). Additionally, the Delta IOP for treated eyes (current IOP minus baseline IOP) was significantly lower than that of the sham‐treated eyes for 43 days (*p* < 0.01).

We wondered whether the extent or duration of IOP reduction might be improved by injecting the HA at two separate locations in each eye. In this study, the total volume of HA injected was held constant, so that 25 µL of HA was injected at each of the two injection sites in the double‐injected eyes, whereas 50 µL of HA was injected in the single‐injection eyes described above. Similar to the situation with a single injection, IOP after double HA injection dropped by ≈4 mmHg right after injection, and then steadily increased to baseline values. Analysis by linear regression showed that IOP in the double‐injected eyes was significantly lower than preinjection IOP values for 35 days (*p* < 0.01). Additionally, the Delta IOP for the double‐injected eyes remained significantly lower than that of the sham group for 45 days (*p* < 0.01). These findings further confirm the hypothesis that SCS injection of gel can reduce IOP for an extended period of time. When comparing the single‐injected to the double‐injected eyes, we found that there were significant differences in IOP between days 8 and day 28 (*p* < 0.01), although the magnitude of this difference was small (<0.6 mmHg) and thus likely clinically unremarkable.

### 2.3 An Optimized Crosslinked HA Formulation Better Resisted Degradation

To further extend IOP reduction, we created new HA hydrogel formulations designed to resist degradation, with the goal of sustaining SCS expansion and IOP reduction. We accomplished this by adding thiol groups to the HA (HA‐SH) and co‐formulating with polyethylene glycol diacrylate (PEGDA) as a cross‐linker. As a baseline measurement, we assayed the degradation rate of the commercial HA‐based hydrogel, which contained 2.4% (w/v) HA with 1,4‐butanediol diglycidyl ether cross‐linker, and found that it dissolved within 2 days in a hyaluronidase solution in vitro (Figure S1A, Supporting Information). We also prepared HA solutions without a crosslinking agent and found that gels composed of 4% (w/v) HA or 8% (w/v) HA dissolved within 2 or 4 days, respectively, when incubated with hyaluronidase (Figure S1B,C, Supporting Information).

In contrast, crosslinked gels created using HA‐SH and PEGDA (i.e., HA‐XL) persisted for more than one month in the hyaluronidase solution. Complete degradation occurred in the less‐crosslinked gel after 33 days (5% (w/v) PEGDA, Figure S1D, Supporting Information) and in the more‐crosslinked gel after 40 days (9% (w/v) PEGDA, Figure S1E, Supporting Information). While we did not expect quantitative equivalence of gel degradation rates in hyaluronidase solution in vitro and in the SCS of the rabbit eye in vivo, these data suggested that the HA‐XL formulations would persist in the eye for much longer than the commercial HA formulation, and thereby provide prolonged IOP reduction.

### 2.4 SCS Injection of Crosslinked HA Formulation Lowered IOP for Four Months Compared to Baseline

We found that a single SCS injection of the more‐crosslinked HA‐XL gel formulation in normotensive rabbits led to an IOP reduction of ≈4 mmHg immediately after injection, which then extended to an IOP drop of ≈6 mmHg over the following 3 days (Figure 2B). IOP then steadily increased over time and approached baseline levels. Statistical analysis showed that mean IOP in the eyes receiving HA‐XL gel injection, as predicted by linear regression, was significantly lower than preinjection IOP values for 119 days after injection (*p* < 0.01). Additionally, Delta IOP was significantly lower than in the sham group throughout the 121 day measurement period (*p* < 0.01). These data indicate that the injection of the HA‐XL gel formulation into the SCS was able to produce an IOP reduction for 4 months.

### 2.5 IOP Reduction Correlated with SCS Expansion

Our hypothesis is that reduced IOP is associated with the expansion of the SCS and that greater SCS expansion correlates with greater IOP reduction. Consistent with this hypothesis, we found that when IOP reduction was greatest (i.e., right after SCS injection of HA‐XL gel), expansion of the SCS measured by ultrasound biomicroscopy was also greatest (Figure 2C). The initial increase in IOP reduction from ≈4 to ≈6 mmHg during the first week after injection corresponded to an increase in eye wall thickness of ≈4 to ≈5 mm. The measured increase in eye wall thickness is interpreted as an increase in SCS thickness since the ocular tissues are not expected to expand (or contract) due to HA‐XL injection, but the SCS is expected to widen. After the first week, as IOP reduction steadily decreased, SCS expansion similarly steadily decreased until 120 days, when there was no longer a significant IOP reduction, and the SCS appeared to be fully collapsed. Statistical analysis showed a quantitative correlation between the amount of IOP reduction and the change in eye wall thickness (linear regression, *R* ^2^ = 0.726) (Figure 2D). According to this correlation, each increase in eye wall thickness of ≈500 µm corresponded to a reduction of ≈1 mmHg IOP in normotensive rabbits.

**Figure 3:**
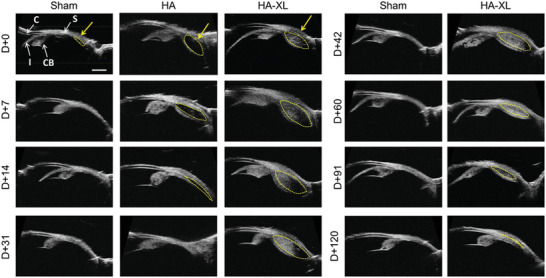
Ultrasound biomicroscopy imaging of hydrogel‐injected eyes. Rabbit eyes were injected with Hanks’ Balanced Salt Solution (Sham), commercial hyaluronic acid hydrogel (HA), or in situ‐forming hyaluronic acid hydrogel group (HA‐XL) and imaged over time. The yellow arrow indicates the approximate injection site, and the yellow dashed line roughly outlines the expanded suprachoroidal space. These images were used to determine eye wall thickness in Figure 2. Images are representative of seven eyes per group (HA‐XL group), two eyes per group (Sham), or the only eye available from the HA group. Abbreviations‐ C: Cornea; CB: Ciliary Body; I: Iris, S: Sclera. D+0 refers to day zero after injection; D+7 to 7 days after, etc. Scale bar: 2 mm.

Expansion of the SCS after injection of HA was examined in greater detail by ultrasound biomicroscopy (**Figure** **3**). Imaging showed an expanded anterior SCS immediately behind the ciliary body after SCS injection. Sham injection of HBSS caused only minor SCS expansion 10 min after injection and was no longer evident after that. After injection of commercial HA gel, the SCS also expanded but collapsed almost to baseline thickness within 14 days, with no expansion visible 31 days after injection.

After injection of HA‐XL gel, SCS expansion was evident 10 min after injection and grew in size during the first week, which we attribute to initial gel swelling as it imbibed water from neighboring tissues. The SCS then remained expanded for at least 91 days (although less expanded at later times) and appeared fully collapsed by 120 days after injection. SCS expansion appeared to be highly localized to the anterior SCS, extending from the anterior end of the SCS adjacent to the ciliary body to as far as 4–5 mm posteriorly. The expansion of the SCS appeared up to ≈3 mm thick at its peak. These findings support the hypothesis that IOP reduction is correlated with SCS expansion.

### 2.6 SCS Injection of Crosslinked HA Appeared Safe

**Figure 4:**
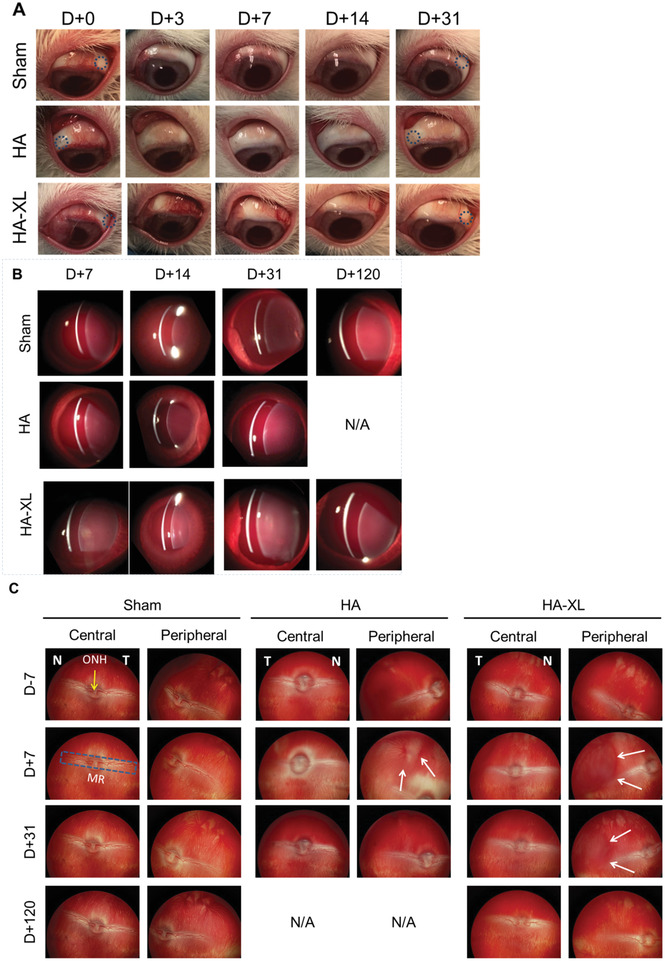
Clinical evaluation of hydrogel‐injected eyes. Rabbit eyes were injected with Hanks’ Balanced Salt Solution (Sham), commercial hyaluronic acid hydrogel (HA), or in situ‐forming hyaluronic acid hydrogel (HA‐XL) and imaged over time. A) Representative images of external features of the eye after suprachoroidal injection. The blue circle on selected images indicates the approximate location of the injection site. B) Representative slit‐lamp biomicroscopy used to assess possible anterior chamber flare in injected eyes. No flare was observed. C) Representative funduscopy was used to identify possible changes in retinal morphology due to injection. The white arrows on selected images indicate the margin of the expanded suprachoroidal space due to the hydrogel. Images are representative of seven eyes per group (HA‐XL group), two eyes per group (Sham), or the only eye available from the HA group. Scale bar: 2 mm. Abbreviations‐ N/A: Not available, T: Temporal, N: Nasal, ONH: Optic nerve head (yellow arrow), MR: Medullary ray (blue box). Other notation as in Figure 3.

During the course of these studies, the rabbit eyes underwent periodic clinical examinations to assess the safety of the SCS injection of HA gels. After the sham injection of HBSS or the injection of commercial HA, eyes did not show any remarkable changes in their external features immediately after injection or over time (**Figure** **4**A). After HA‐XL injection, eyes appeared normal immediately after the injection, but by day 3 after injection, eyes presented with mild to moderate surface hyperemia at the site of injection, indicating localized irritation (Figure 4A). The hyperemia lessened over time and later fully resolved within 1–2 weeks. Because the commercial HA gel did not have this effect, we attribute the irritation to the PEGDA crosslinking agent, which may need further optimization in terms of concentration, extent of preinjection crosslinking, and other parameters. All other external features in the clinical exam appeared normal.

Slit‐lamp biomicroscopy exams appeared normal during all exams for the Sham, HA gel, and HA‐XL gel formulations (Figure 4B). There was no evidence of flare (indicating inflammation in the anterior segment), which suggested that the hyperemia seen in the clinical exam was localized to the ocular surface and did not induce uveitis. Also, no significant structural abnormality was observed by ultrasound biomicroscopy in any group of animals (Figure 3).

Fundoscopy was also generally unremarkable (Figure 4C). Retinas and posterior structure of the rabbit eyes were clearly visible, and there were no floating particles or layers in the posterior eye that suggested evidence of retinal detachment, tears, hemorrhage, hydrogel leaking, or vitreous hemorrhage. However, the peripheral retina of eyes receiving either HA or HA‐XL gel appeared to be “bulging out.” This retinal deformation was first seen one week after injection and was found to resolve over time (Figure 4C, white arrows). We attribute this bulging appearance to the presence of gel expanding the SCS, which provides further support for our expectation that the injection of gel into the SCS expands SCS thickness.

**Figure 5:**
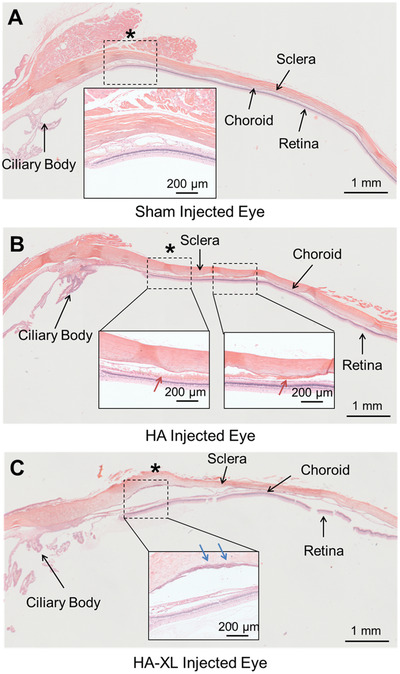
Histopathological analysis of hydrogel‐injected eyes. Rabbit eyes were injected with A) Hanks’ Balanced Salt Solution (Sham), B) commercial hyaluronic acid hydrogel (HA), or C) in situ‐forming hyaluronic acid hydrogel (HA‐XL). Red and blue arrows indicate local hemorrhage and fibrotic tissue formation, respectively. * is the approximate site of the injection. Images are representative of seven eyes per group (HA‐XL group), two eyes per group (Sham). Only one eye was available from the HA group. Scale bars: 1 mm.

At the end of the study, i.e., 121 days after injection, the eyes were enucleated and subjected to histopathological examination (**Figure** **5**). Tissues in sham‐injected eyes did not show any significant abnormalities (Figure 5A). However, evidence of local (non‐expulsive) hemorrhage was found in the rabbit eye that had received HA gel (Figure 5B). Near the injection site of eyes receiving HA‐XL gel, eyes exhibited mild immune cell filtration, minimal to moderate fibrotic formation, and an enlarged suprachoroidal space, which suggests that the extended presence of HA‐XL gel in the SCS generated a local immune response (Figure 5C). Additional studies are needed to determine the clinical significance of these findings.

Although this study was designed to lower IOP, immediately after injection of Sham, HA, or HA‐XL formulations, there was a transient increase of IOP, which subsequently dropped to baseline within one hour (Figure S2, Supporting Information). This behavior is consistent with prior studies of SCS injection of other formulations^[^ ^15^ ^]^ and is believed to be due to the increase of intraocular volume caused by the introduction of fluid into the eye (i.e., in this case, 50 µL of fluid) followed by a return to baseline pressure as the fluid is cleared from the eye (e.g., via choroidal vessels and/or across the sclera).^[^ ^22^ ^]^ This is also known to occur routinely after intravitreal injection, where it is generally well tolerated.^[^ ^17^ ^]^

### 2.7 IOP Reduction Is Not Due to Changes in Conventional Outflow

**Figure 6:**
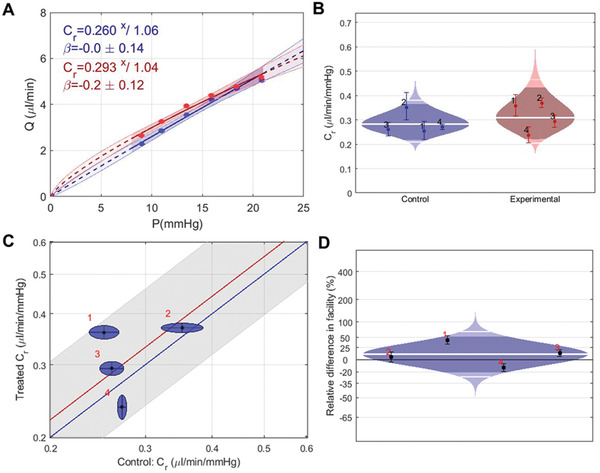
Measurements of aqueous outflow facility to identify the mechanism of IOP lowering after injection of in situ‐forming hydrogel (HA‐XL). A) A representative flow–pressure (Q–P) trace for an eye receiving HA‐XL (red) and the contralateral naïve control eye (blue). Data points are fitted with an existing relationship (solid lines) with associated 95% confidence limits (shaded region), as previously described,^[^ ^44^ ^]^ and used to obtain a conventional outflow facility, *C_r_*, at a reference pressure of 8 mmHg. B) The data is presented in a “cello plot,” showing the distribution of reference facilities in eyes receiving HA‐XL (red, *n* = 4) and naïve contralateral control eyes (blue, *n* = 4). Each data point shows the reference facility for one eye, with the error bars showing 95% confidence intervals. Shaded regions show the best estimates of the sample distributions, with the geometric mean and two‐sigma shown by the thick and thin horizontal lines, respectively. Dark central bands show the 95% confidence interval on the mean. C) A “unity plot” for reference outflow facility, in which facility in the treated eye is cross‐plotted against the facility in the contralateral control eye. Each point represents one animal, with ellipses indicating 95% confidence intervals from the regression fitting. The unity line is shown in blue, and a regression to the data (in the log‐transformed domain^[^ ^44^ ^]^) is shown in red with the 95% confidence interval in grey. D) The HA‐XL injection leads to a mild (10%) increase in reference outflow facility, which is not statistically significant (*p* = 0.41, paired t‐test on log‐transformed data with threshold *p* = 0.05). The plotted quantity is the percentage difference between contralateral eyes, and the plot is otherwise interpreted as in panel B. Panels (B), (C), and (D) show the same data plotted in different ways.

To further understand the effects of injected HA hydrogel on aqueous humor dynamics, we measured conventional outflow facility using iPerfusion, an ex vivo method to determine the resistance to fluid flow out of the eye as a function of IOP (**Figure** **6**). One week after injecting HA‐XL gel into the SCS of rabbits, we euthanized the animals and measured outflow facility in enucleated eyes. We observed no significant difference (*p* = 0.41) of outflow facility between control (0.28 ^×^/1.30 µL min^−1^ mmHg^−1^) and HA‐XL hydrogel‐injected eyes (0.31 ^×^/1.40 µL min^−1^ mmHg^−1^), which means that hydrogel injection into the SCS did not appear to significantly affect aqueous outflow dynamics. Since this technique specifically measures conventional outflow facility through the trabecular meshwork, this finding supports our hypothesis that increased uveoscleral outflow via the unconventional pathway was responsible for lower IOP in hydrogel‐treated eyes.

## 3 Discussion

In this study, we demonstrated that a single SCS injection of an in situ‐forming hydrogel reduced IOP in the normotensive rabbit for four months without significant complications. This procedure suggests a new strategy for IOP control, hypothesized to involve increased uveoscleral outflow by the mechanical expansion of the SCS. The duration of the IOP‐lowering effect was extended by optimizing the hydrogel formulation to increase crosslinking and thereby slow HA degradation and clearance rate.

### 3.1 Translational Considerations

The development of a method to control IOP without surgery or drugs would be a major advance since existing treatments are associated with significant side effects and waning efficacy. Surgical procedures involve the creation of artificial aqueous humor outflow pathways, either with or without (e.g., trabeculectomy) implantation of a device. In either case, surgical penetration of the globe is required, which is associated with a risk of complications, including hyphema (blood in the anterior chamber), corneal edema (swelling of the cornea), blebitis (infection of a bleb in the eye), endophthalmitis (severe ocular inflammation usually due to infection), and phthisis (shrunken eye with little or no function).^[^ ^5^, ^23^ ^]^ Often, scar tissue forms that block the newly created pathways for aqueous humor drainage; this and other complications mean that trabeculectomy has a relatively high failure rate (7% and 15% at 10 and 20 years, respectively), necessitating re‐operation.^[^ ^24^ ^]^ Even with surgery, vision loss progresses within 5 years in 30% of patients.^[^ ^25^ ^]^ Finally, the surgical treatment of glaucoma is expensive, with the 5‐year cumulative cost of trabeculectomy being reported as $6363 per patient in 2008.^[^ ^26^ ^]^ While surgical implantation of a drainage device usually has a lower complication rate compared to trabeculectomy, it is much more costly and has a failure rate no better than trabeculectomy.^[^ ^27^ ^]^

Pharmaceutical treatment of glaucoma can have ocular and systemic adverse effects, in addition to suffering from a significant lack of patient adherence.^[^ ^3^ ^]^ Among the most commonly prescribed medications, prostaglandin analogs suffer from side effects, e.g., change of iris color and growth of eyelashes; alpha‐adrenergic receptor agonists can cause oral dryness, ocular hyperemia, and ocular allergy; beta‐blockers are associated with cardiovascular side effects if systemic exposure occurs; and carbonic anhydrase inhibitors can cause ocular stinging, burning, itching, and bitter taste. In addition, glaucoma medications often lose efficacy over time so that patients often need to change medications or combine multiple drugs.^[^ ^3^ ^]^ Moreover, the cost of glaucoma drugs is substantial (e.g., $150–875 per drug per year).^[^ ^28^ ^]^

Unlike surgery, we expect that SCS injection of HA‐XL gel can be performed as an office procedure since SCS injections using microneedles have been performed in an office setting in a number of different clinical trials without significant complications.^[^ ^29^ ^]^ The injection is minimally invasive because the hydrogel is able to flow through a microneedle and only complete gelation once in the SCS, which should reduce the risk of complications, shorten procedure and recovery time, and reduce cost compared to surgery. Unlike pharmaceuticals, SCS injection of HA‐XL would not require daily adherence and would not have the off‐target pharmacological effects often associated with drugs.

### 3.2 Mechanistic Considerations

Expansion of the SCS associated with ciliochoroidal detachment during intraocular surgery can cause dramatic IOP lowing (hypotony), and the mechanism has been suggested to be an increase in uveoscleral outflow,^[^ ^30^ ^]^ which is consistent with results from an animal model.^[^ ^31^ ^]^ Guided by these findings, as well as our own prior observations,^[^ ^13^ ^]^ we hypothesized that injection of hydrogel into the SCS could similarly reduce IOP, possibly by facilitating drainage of aqueous humor via the uveoscleral pathway. Because reduced IOP has been seen after SCS expansion without the injection of hydrogel,^[^ ^19^ ^]^ it appears that the opening of the SCS is mechanistically important and that the hydrogel is itself not directly responsible for IOP reduction, but is instead a means to open the SCS.

While the simplest explanation for the observed IOP drop associated with SCS expansion is an increase in aqueous humor outflow via the uveoscleral route, we considered four alternate explanations for the IOP drop. We first considered an increase in conventional (pressure‐dependent) outflow facility via the trabecular meshwork, which is well established to lower IOP.^[^ ^3^ ^]^ However, direct measurements on treated rabbit eyes showed no change in the conventional outflow facility. As a second possible explanation, we considered uveitis induced by hydrogel injection, which could lower IOP.^[^ ^32^ ^]^ However, our clinical evaluation with a slit‐lamp biomicroscope showed no anterior chamber flare in any eye, indicating a lack of inflammation. A third possibility is that a reduction in episcleral venous pressure could lower IOP.^[^ ^33^ ^]^ Although we did not measure blood pressure in this study, a previous study examining acute experimental ciliochoroidal detachment in a monkey model showed no significant differences in episcleral venous pressure between the experimental and the control groups.^[^ ^34^ ^]^ The fourth possibility is that hydrogel injection near the ciliary body could reduce aqueous humor secretion rate, similar to the effects of cyclodialysis.^[^ ^35^ ^]^ Using the data available, we cannot rule out this final alternate explanation, so future studies will need to investigate the role of increased uveoscleral flow in the reduction of IOP seen in this study.

### 3.3 Safety Considerations

Transient SCS expansion (a.k.a. choroidal or ciliochorodial detachment, suprachoroidal, or supraciliary effusion) commonly occurs in clinical practice after an unintended accumulation of fluid in the SCS after glaucoma surgery.^[^ ^35^ ^]^ The cause‐effect relationship between ocular hypotension and suprachoroidal detachment in these cases remains unclear, but the SCS is known to close without lasting adverse effects. This suggests that SCS expansion by hydrogel may similarly be well tolerated, although the mechanisms and durations of suprachoroidal detachment are different. Further studies will be required to evaluate the long‐term effects of SCS expansion by the hydrogel.

In this study, we also found a rapid (<1 h), transient elevation of IOP after hydrogel injection into the SCS. Such transient elevations of IOP have been observed in research studies of SCS injection^[^ ^15^, ^16^ ^]^ and intravitreal hydrogel injection,^[^ ^36^ ^]^ and are a standard feature of conventional, clinical intravitreal injections performed millions of times per year for treatment of age‐related macular degeneration and other indications.^[^ ^17^ ^]^ Since intravitreal injections are well‐tolerated in patients, we expect that the transient elevation of IOP after our procedure would similarly be safe and well‐tolerated in the clinic. Indeed, intravitreal injections with similar transient increases in IOP are not contraindicated for preexisting glaucoma^[^ ^37^ ^]^ and have been used in neovascular glaucoma patients.^[^ ^38^ ^]^ If a major concern exists for a specific patient, paracentesis before SCS injection could be an option to reduce the risk caused by a transient IOP elevation.

### 3.4 Study Limitations

We used the normotensive rabbit eye in this study. To translate our findings to human glaucoma patients, studies should be done on other species and in hypertensive and glaucomatous eyes. A previous study with a rhesus macaque model reported that SCS expansion with Ringer's solution or autologous serum reduced IOP by 3.0–6.5 mmHg for more than 14 days.^[^ ^30^ ^]^ This result in a nonhuman primate suggests that SCS expansion to reduce IOP over a sustained period may also be effective in humans, but further research is needed. Also, a prior study compared the effect of prostaglandin (travoprost), which shares the same general putative mechanism of action with our method, on IOP in normotensive and hypertensive eyes. In normotensive eyes, the resulting pressure drops were 4.0 and 2.7 mmHg at 2.25 and 16 h after drug administration, respectively, while in hypertensive eyes, the drops were 7.9 and 8.8 mmHg at the same time points.^[^ ^39^ ^]^ This suggests that the expected IOP reduction using our approach in hypertensive eyes would exceed the reduction we report herein normotensive eyes. This expectation is also consistent with Goldmann's equation, commonly used to interpret aqueous humor outflow dynamics,^[^ ^40^ ^]^ which predicts that if episcleral venous pressure, aqueous inflow rate, and conventional outflow facility are unaffected by suprachoroidal injection, then the magnitude of IOP reduction is larger in eyes with reduced outflow facility. However, further research is needed to better understand the effects in hypertensive eyes.

Future studies will also be needed to more broadly validate our finding of reduced IOP for months after hydrogel injection into the SCS, ultimately being assessed in human trials. In addition, while no major safety signals were found in this study, a more detailed analysis of safety that includes assessments of visual acuity, electro‐retinal function, and visual fields will be needed in larger animal models, such as the dog, pig, or nonhuman primate, as well as in human subjects. While we observed reduced IOP for four months, improved hydrogel formulations and other changes would be desirable if they extended the period of IOP reduction even longer (e.g., 6 months) and maintained a more constant IOP reduction that does not slowly return to baseline IOP level over the course of the treatment.

IOP could be reduced for longer times using periodic repeat SCS injections. It is possible that local micro‐scale trauma (e.g., hemorrhage) and the immune response that causes fibrosis precludes repeated injections at the same site for safety reasons. In this case, a repeated injection could be performed at different sites around the eye to at least partially extend the treatment duration. Alternatively, local micro‐scale trauma could be reduced by optimizing microneedle design, hydrogel formulation, and/or injection procedure. Further, fibrosis could be alleviated by the addition of antifibrotic agents to the formulation(e.g., anti‐IL 17).^[^ ^41^ ^]^

In the present study, HA formulation and injection procedures were designed to avoid contamination and secondary bacterial infection. However, we were not able to obtain pharmaceutical grade HA‐SH or PEGDA, which should be used in future studies.

Finally, while our data in the context of the broader literature indicate that increased drainage through the unconventional (uveoscleral) outflow route is the mechanism of IOP reduction, we were not able to show this directly. We tried to evaluate uveoscleral outflow by injecting fluorescent tracer into the anterior chamber of live animals' eyes and visualizing its distribution after euthanization. Unfortunately, technical issues prevented us from obtaining reliable information from this small cohort of eyes, and thus further mechanistic studies are indicated.

## 4 Conclusion

This study demonstrated the reduction of IOP for four months by the expansion of the SCS with an in situ‐forming HA hydrogel administered using a microneedle without significant complications. With further research and development, this drug‐free, nonsurgical procedure has the potential to be used for the low‐cost treatment of ocular hypertension and glaucoma without the need for daily patient adherence or invasive surgical interventions.

## 5 Experimental Section

##### Microneedle and In Situ‐Forming Hydrogel Preparation

A 27‐gauge expansion needle (Air‐Tite, Tochigi, Japan) was used to fabricate a hollow microneedle, as previously described.^[^ ^19^ ^]^ Briefly, after shortening the needle to ≈1.0 mm in length using a conventional cutter, the needle was ground with a cordless rotary tool (Dremel 800, Robert Bosch, Gerlingen, Germany). The length of the needle was measured to be ≈650–750 µm under a stereomicroscope, and the needle was then sterilized using ethylene oxide (Anprolene AN74j sterilizer, Andersen Products, Haw River, NC). An in situ‐forming crosslinked hydrogel (HA‐XL) was prepared using 3% (w/v) thiol‐modified hyaluronic acid (HA‐SH) (Glycosil, ESI Bio, Alameda, CA) and 5% or 9% (w/v) poly(ethylene glycol) diacrylate (PEGDA, MW 3500 Da, JenKem Technology, Beijing, China) dissolved in HBSS. While both the 5% and 9% PEGDA formulations were studied in vitro, only the 9% PEGDA formulation was used in vivo. For the in vivo study, aliquots of HA‐SH and PEGDA were prepared in a conventional biosafety cabinet, and sterile HBSS was used to dissolve all compounds. After 15 min at room temperature (20–25 °C), the HA‐XL hydrogel was loaded in a syringe (1 mL Luer‐lock plastic syringe; BD Bioscience, San Jose, CA) following aseptic technique, and the microneedle was attached.

##### Animals

Eleven New Zealand White rabbits (either sex, 3.0–3.5 kg) were purchased from Charles River Breeding Laboratories (Wilmington, MA). All animal studies were carried out in accordance with the Association for Research in Vision and Ophthalmology (ARVO) Statement for the Use of Animals in Ophthalmic and Visual Research, and all experimental procedures were approved by the Georgia Institute of Technology Institutional Animal Care and Use Committee. The rabbits were housed individually at a controlled temperature of 19.3 ± 0.6 °C and humidity of 61.4 ± 7.1% on a 12:12 hour light:dark cycle. After a one week acclimation period, all eyes were confirmed to be clinically normal by an ophthalmic examination before studies were begun.

Three sets of animal studies were performed. In the first, 50 µL of a commercial HA hydrogel (2.4% (w/v) HA, 6% crosslinked, Juvederm Ultra XC, Allergan, Irvine, CA) was injected into the SCS in two animals to explore the effects of suprachoroidal expansion on IOP (Figure 1C). In each animal, one eye received 50 µL of the commercial HA solution injected in the superotemporal quadrant of the eye, while the other eye received 25 µL of the commercial HA solution injected in the superotemporal quadrant and 25 µL of the commercial HA solution injected in the superonasal quadrant. Both rabbits were euthanized 50 days after the injection.

In the second study, five animals were used to evaluate the safety and efficacy of an in situ‐forming crosslinked HA hydrogel (HA‐XL, see below) expected to extend the duration of IOP reduction. In this second cohort, seven eyes received 50 µL of the HA‐XL formulation injected into the SCS, two eyes received a sham injection of 50 µL of Hanks’ Balanced Salt Solution (HBSS, Gibco, Grand Island, NY), and one eye was injected with 50 µL of the commercial HA formulation (Figure 1D). In this experiment, the HA‐SH and PEGDA were mixed for 15 min before injection to initiate the crosslinking reaction and then injected into the SCS where the crosslinking was completed in situ in the eye. The eye receiving the commercial HA formulation was used only for clinical evaluations for 31 days because IOP returned to baseline well before that time. All injections in the second study were performed in the superotemporal quadrant of the eye. Animals in the second study were euthanized 121 days after injection, and the collected specimens were processed for histopathological examinations.

In the third study, four animals were used for measuring aqueous outflow facility. Each rabbit received 50 µL of the HA‐XL formulation injected into the SCS in the superotemporal quadrant of a randomly chosen eye. The other eye served as a naïve control without treatment. Animals in this cohort were euthanized 7 days after injection (Figure 1E), and the outflow facility was measured in the enucleated eyes, as described below.

At the end of all studies, rabbits were euthanized under general anesthesia using a combination of ketamine (25 mg kg^−1^) and xylazine (5 mg kg^−1^) with an intravenous injection of euthanasia solution (1.5 mL per animal, Euthasol, Virbac, Fort Worth, TX).

##### Suprachoroidal Injection

General anesthesia was induced using an induction chamber (Model 90 100, Bickford, Wales Center, NY) with 5% isoflurane (Isothesia, Henry Schein, Dublin, OH) and a 400 mL min^−1^ oxygen flow rate for 7.5 min. Following induction, anesthesia was maintained with 2–3% isoflurane, which was found to lead to transient ocular hypertension (18–30 mmHg). Before injection, the eye was irrigated with sterile saline, and then 5% of a povidone‐iodine solution (Betadine, Alcon, Fort Worth, TX) was instilled into the eyes for sterilization. After applying two drops of topical ophthalmic anesthetic (Proparacaine, Akron, Lake Forest, IL), either hydrogel or Sham HBSS (i.e., HBSS without HA or PEGDA) was injected into the SCS of each eye with a hollow microneedle at a location 2–3 mm posterior to the limbus. After the injection, successful SCS expansion was confirmed by ultrasound biomicroscopy, as described below, and an antibacterial ointment (Neomycin, Polymyxin B sulfate and Bacitracin zinc ophthalmic ointment USP, Bausch + Lomb, Rochester, NY) was applied to prevent secondary infection.

##### Tonometry and Clinical Evaluations

IOP was measured using a hand‐held tonometer (TonoVet, iCare, Espoo, Finland) every day between 10 AM and 12 noon without the use of chemical agents or restraint. Recorded values were the average of five consecutive measurements. The baseline IOP value for each eye was taken as the average over five to seven days before the SCS injection for each animal within each treatment group. The measurement of the baseline IOP for each animal and each time point is shown in Figure S3 in the Supporting Information. Tonometric IOP measurements (IOP_ton_) were converted to actual IOP (IOP_act_) using a relationship obtained from a calibration study that was performed before:^[^ ^42^ ^]^ IOP_actual_ = 0.8784 IOP_tonometric_ − 1.26 (IOP in units of mmHg). Briefly, A 25‐gauge needle was inserted into the anterior chamber of five fresh ex vivo rabbit eyes. A reservoir was connected to the needle containing a balanced salt solution that was elevated to different heights to set IOP. The measurements by tonometry were produced as IOP was set at levels from 5–20 mmHg in increments of 5 mmHg, which were used for obtaining a calibration curve.

Clinical evaluations were performed using three ophthalmic instruments under general anesthesia by a veterinary ophthalmologist who prepared materials, preformed the injection, and measured the IOP of rabbits. For preanesthesia, the animal received a mixture of ketamine (10 mg kg^−1^, Ketathesia, Henry Schein) and xylazine (2 mg kg^−1^, AnaSed Injection, Akron), after which anesthesia was maintained with 1.5–3.0% isoflurane. First, an ultrasound biomicroscope (UBM Plus, Accutome, Malvern, PA) was used to image the SCS and surrounding ocular tissue structures. Serial images were acquired 10 min and 7, 14, 31, 42, 60, 91, and 120 days after the SCS injection. After attaching a sterile probe cover (ClearScan, Eye‐Surgical‐Instruments, Plymouth, MN), the ultrasound probe was placed over the injection site. The maximum eye wall thickness (i.e., from the external conjunctival surface to the internal limiting membrane of the retina, including hydrogel in the SCS) at the injected site was calculated by averaging measurements obtained from three different images per time point using software bundled with the ultrasound imaging instrument (Accutome connect 8.02.02). Baseline values of eye wall thickness were determined from images acquired from the noninjected equivalent area (i.e., 3 mm posterior to the limbus from the inferonasal quadrant of the eye).

Second, conventional examinations of the anterior eye were conducted using a slit‐lamp (Model 253, Mentor, Tokyo, Japan) with a standard table at 7, 14, and 31 days after the suprachoroidal injection. Third, the posterior eye was examined by instilling two drops of tropicamide (Henry Schein) and phenylephrine (Henry Schein) at 5‐min intervals to dilate the pupil. Fundus images were obtained using a fundus scope (Reticam II, Clarity Medical Systems, Pleasanton, CA) attached to a 130° lens 7 days before and 7, 31, and 120 days after the SCS injection in a dark room.

Finally, photographs of the external features of the eyes were taken 1 h, and 3, 7, 14, and 31 days after the injection.

##### Histopathological Examination

Eyes from the second study were enucleated, fixed with Davidson's solution (Electron Microscopy Science, Hatfield, PA) and 10% formalin, dehydrated, and embedded in paraffin. Histological sections were cut using a rotary microtome (HM 355 S, Thermo Fisher Scientific, Waltham, MA) and stained with either hematoxylin and eosin (H&E), using a standard procedure.^[^ ^43^ ^]^ The stained slides were imaged by brightfield light microscopy (Axio Observer Z1, Zeiss). More than four images for each group were evaluated by an ophthalmic pathologist masked as to treatment protocol.

##### Outflow Facility Measurement

Four pairs of eyes from the third study were enucleated 7 days after injection and mounted on eye holders in an iPerfusion system, which comprises an actuated pressure reservoir, a pressure transducer (PX409, Omegadyne, USA) and a thermal flow meter (SLI 0150, Sensirion, Switzerland), as previously described in detail.^[^ ^44^ ^]^ Enucleated eyes were cannulated with a 27‐gauge 0.5‐inch length sterile needle (Becton Dickenson, Franklin Lakes, NJ) and perfused under a steady pressure of 11 mmHg for 30 min with DBG solution (1 × Dulbecco's phosphate‐buffered saline with 5.5 × 10^−3^ m glucose). Ten sequential pressure steps of 7, 9, 11, 13.5, 16, 18.5, 21, 16, 12, and 8 mmHg were then applied to eyes by adjusting the height of the actuated reservoir. Flow rate (Q) and pressure (P) were recorded continuously until steady‐state was reached, defined as the slope of the measured flow rate versus time being less than 40 nL min^−1^ min^−1^ for 1 min. Here, the slope was calculated from the change in recorded flow rate over a 300‐second window, divided by 300 s. After the rejection of outlier steps, the outflow facility was calculated from fitting flow–pressure data at 9, 11, 13.5, 16, 18.5, and 21 mmHg using a power law, as previously described in detail.^[^ ^44^ ^]^ Because outflow facility is log‐normally distributed, the variability of the data is using a multiplicative standard deviation, denoted by ^×^/.^[^ ^44^ ^]^

##### Statistical Analysis and Data Presentation

All values are presented as mean ± standard deviation (S.D.). The first and second studies were statistically analyzed to evaluate the efficacy of each treatment. The number of samples in the HA‐XL group was seven, and that of the other groups was two. Mean and 95% confidence intervals for baseline IOP values were calculated by combining all baseline measurements (across animals and days) within each treatment group. Least squares linear regression was used to model IOP versus time post‐injection in GraphPad Prism software (version 8.0.0 for Windows, GraphPad Software, San Diego, CA). For example, in the HA‐XL group, linear regression was performed using the 7 eyes in that group, comprising 121 data points at different time points for each eye, meaning that the mean and 95% confidence bands calculated for this group were derived from 847 data points. The null hypothesis for the analysis of regressions was that one regression line adequately fit all treatment groups, with the threshold significance for the rejection of this hypothesis taken as *p* = 0.05. We found evidence to reject this hypothesis using GraphPad's extra sum‐of‐squares F test (*p* < 0.0001), substantiating the use of individual regression lines for each group. Mean and 95% confidence intervals for baseline IOP were compared to the mean and 95% confidence bands of the relevant treatment group's regression line. The time points at which statistically significant differences in IOP existed were determined using the intersection of 95% confidence intervals of the baseline IOP and 95% confidence bands of the treatment group IOP. It was noted that the important distinction that the “95% confidence interval” was an indication of the certainty associated with the mean of the baseline values while the “95% confidence band” was an indication of the certainty associated with the location of the post‐injection regression line. The intersection of 95% confidence intervals (for baseline means) or bands (for regression lines) was known to correspond with an approximate p‐value of *p* = 0.01^[^ ^45^ ^]^ for the test of differences in parameters compared and was taken as the threshold for statistical significance for 1) comparison of values of two regression lines at every time point or 2) comparisons between a regression line value at every time point and the respective baseline mean. The time points at which significant differences occurred for compared values are reported.

In addition to comparisons of each treatment group's regression line to baseline IOP, Delta IOP was computed as the post‐injection IOP minus that eye's baseline average IOP, regressed Delta IOP on time post‐injection, and compared regression lines of between treatment groups and the sham group. Additionally, regression lines were compared between the Single and Double HA injection groups. The 95% confidence bands were calculated for each regression line, and statistically significant differences were determined using the intersection of 95% confidence bands between compared regression lines, as previously stated. Mean values were calculated from the regression line for each treatment group.

The normality of residuals for regression lines was tested using the D'Agostino‐Pearson omnibus (K2) test, and the homoscedasticity of residuals was assessed using the test for homoscedasticity function in GraphPad. Analyses of residuals are reported in Figures S4–S7 in the Supporting Information.

To analyze differences in outflow facility between control (*n* = 4) and experimental (*n* = 4) paired eyes, a paired t‐test was performed on log‐transformed data using MATLAB (Version 9.7.0 for Windows, Mathworks, Natick MA). The threshold for statistical significance was taken as *p* = 0.05.

## Conflict of Interest

M.R.P. serves as a consultant to companies, is a founding shareholder of companies, and is an inventor on patents licensed to companies developing microneedle‐based products (Clearside Biomedical). These potential conflicts of interest have been disclosed and are managed by Georgia Tech. J.J.C., J.H.J., C.R.E., and M.R.P. are listed as co‐inventors on an IP filing related to the above study.

## Supporting information

Supporting Information
