# Supplementary material for: Drug‐Free, Nonsurgical Reduction of Intraocular Pressure for Four Months after Suprachoroidal Injection of Hyaluronic Acid Hydrogel
Source: Adv Sci (Weinh). 2020 Dec 7;8(2):2001908. doi: 10.1002/advs.202001908 (PMC7816721; doi:10.1002/advs.202001908)
Supplement: Supplementary file 1 — Supporting Information [file ADVS-8-2001908-s001.pdf]

© 2020 Wiley-VCH GmbH

ADVANCED SCIENCE
Open Access

Supporting Information

for *Adv. Sci.*, DOI: 10.1002/advs.202001908

Drug-free, Non-surgical Reduction of Intraocular Pressure for Four Months after Suprachoroidal Injection of Hyaluronic Acid Hydrogel

J. Jeremy Chae, Jae Hwan Jung, Wei Zhu, Brandon G. Gerberich, Mohammad Reza Bahrani Fard, Hans E. Grossniklaus, C. Ross Ethier, and Mark R. Prausnitz**

WILEY-VCH

© 2020 Wiley-VCH GmbH

Supporting Information

Drug-free, Non-surgical Reduction of Intraocular Pressure for Four Months after Suprachoroidal Injection of Hyaluronic Acid Hydrogel

J. Jeremy Chae, Jae Hwan Jung, Wei Zhu, Brandon G. Gerberich, Mohammad Reza Bahrani Fard, Hans E. Grossniklaus, C. Ross Ethier, and Mark R. Prausnitz**

Supporting Information

Drug-free, Non-surgical Reduction of Intraocular Pressure for Four Months after Suprachoroidal Injection of Hyaluronic Acid Hydrogel

J. Jeremy Chae, Jae Hwan Jung, Wei Zhu, Brandon G. Gerberich, Mohammad Reza Bahrani Fard, Hans E. Grossniklaus, C. Ross Ethier, and Mark R. Prausnitz**

Supplementary Materials & Methods

The *in situ*-forming hyaluronic acid (HA) hydrogel formulation was optimized using a modified hyaluronidase degradation test. Three HA hydrogel constructs were placed into, or cast using, a cap from a 2-ml Eppendorf tube with one of the following five formulations: commercial HA (Juvederm Ultra XC, Allergan, Irvine, CA); 4% or 8% (w/v) non-crosslinked HA (sodium hyaluronate 5 kDa, Lifecore Biomedical, Chaska, MN) dissolved in Hank's Balanced Salt Solution (HBSS); or *in situ*-forming hydrogels with 3% (w/v) thiol-modified HA and either 5% or 9% (w/v) poly(ethylene glycol) diacrylate dissolved in HBSS. Each hydrogel construct was immersed in 2.5 ml of an aqueous solution containing 10 U hyaluronidase (Hyaluronidase from bovine testes, Sigma Aldrich, St. Louis, MO). After removing the solution by wicking into filter paper, images were taken of the remaining hydrogel before and 1, 3, 7, 14, and 31 days after starting the degradation test. If there was no visible hydrogel left in the tube, it was assumed to be fully degraded.

Acute changes in intraocular pressure (IOP) were measured before and after injections of 50µl of commercial hyaluronic acid (HA, n = 2 eyes) or *in situ*-forming HA hydrogel (HA-XL, n = 5 eyes). After initiating inhalation isoflurane anesthesia, which induced transient ocular hypertension, baseline IOP under anesthesia was measured 7.5 minutes after the start of the maintenance period and then again before injection of the hydrogel. IOP was also measured immediately, 5, 10, 15, 30, 45, and 60 min after the hydrogel injection. Inhalation

anesthesia was maintained during IOP measurements, and the concentration of the isoflurane (2-3%) was not changed for 60 min after the injection.

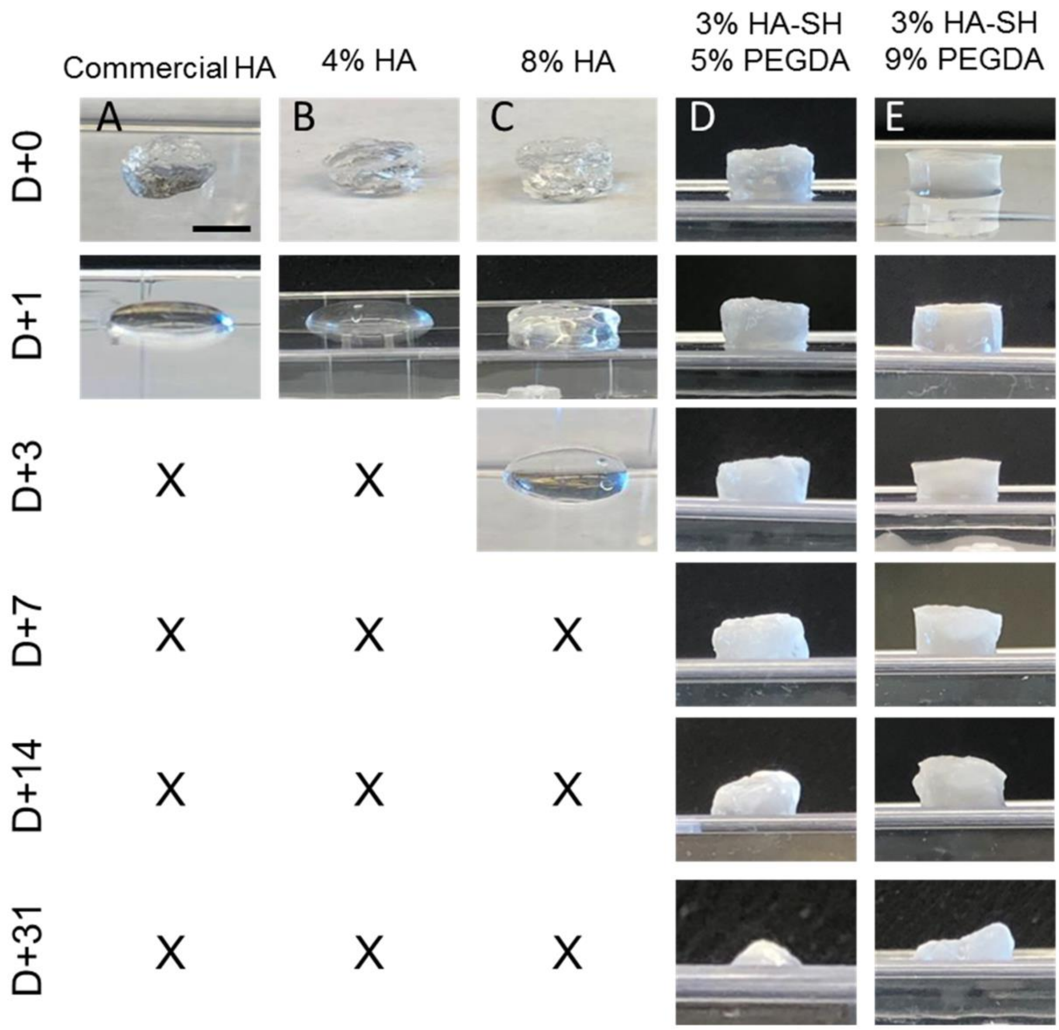

Figure S1. Degradation characteristics of hyaluronic acid (HA) formulations. A hydrogel construct for each formulation was degraded in 2.5 ml of an aqueous solution containing 10 U hyaluronidase. Hydrogel formulations were: commercial HA, 4% and 8% (w/v) HA, and *in situ*-forming crosslinked HA hydrogel (HA-XL) formed by combining 3% (w/v) thiol-modified HA (HA-SH) and either 5% or 9% (w/v) poly (ethylene glycol) diacrylate (PEGDA). Scale bar: 5 mm. Figures are representative of three hydrogel constructs for each group. X indicates that the construction could not be seen and thus was assumed to be fully digested. Abbreviations- D: day. D+0 refers to day zero, i.e., immediately after starting degradation; D+7 refers to 7 days after, etc.

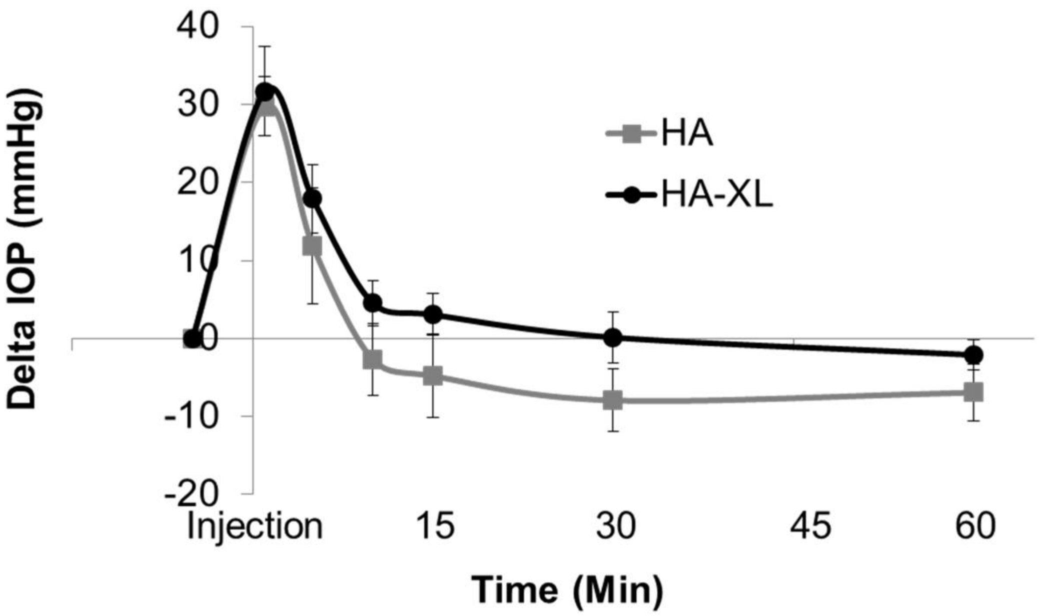

Figure S2. A transient increase in intraocular pressure (IOP) was observed after suprachoroidal injection. We plot delta IOP (change in IOP compared to the baseline IOP under the general anesthesia) vs. time after suprachoroidal injection of commercial hyaluronic acid (HA) and in situ-forming HA hydrogel (HA-XL). Results are presented as mean ± standard deviation from five eyes (HA-XL group) or two eyes (HA group) per group.

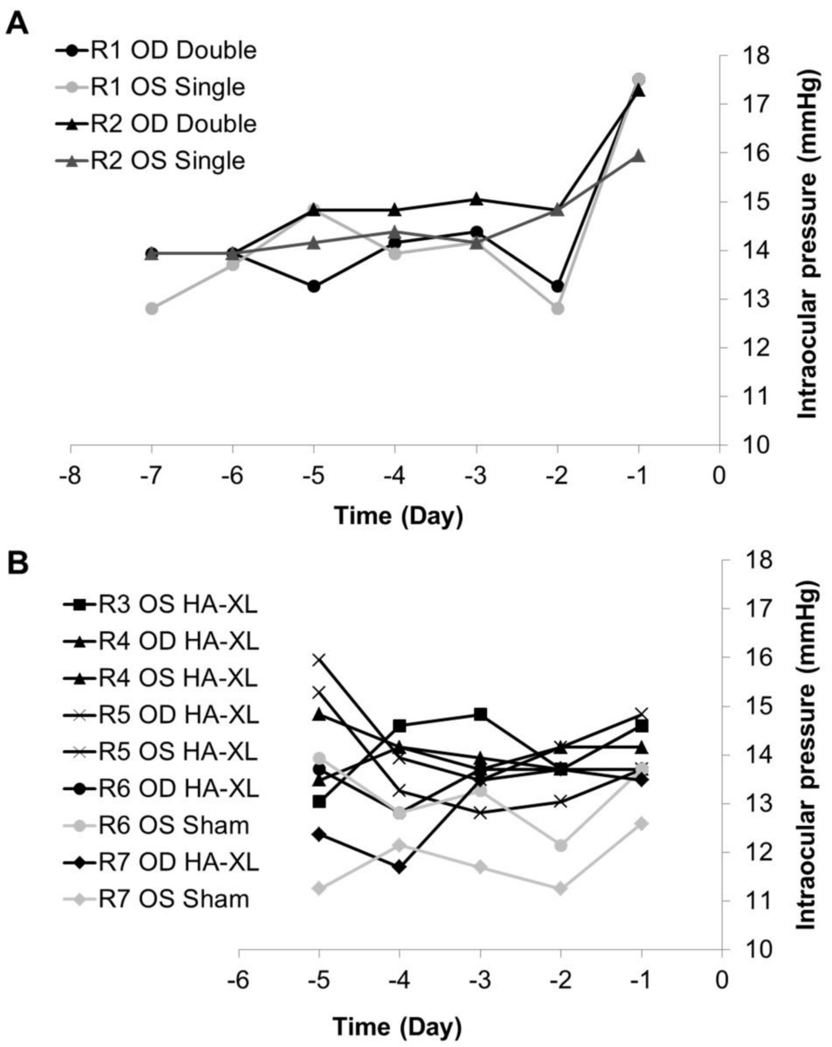

Figure S3. Intraocular pressure (IOP) variation of the baseline measurement for (A) the first study and (B) the second study before the injection. The x-axis shows days before hydrogel injection. After the baseline IOP measurement, each eye received a commercial hyaluronic acid (HA) hydrogel at one site (Single group) or two sites (Double group), an *in situ*-forming HA hydrogel (HA-XL group), or Hanks' Balanced Salt Solution (Sham group) at one site. Abbreviations- R: Rabbit identification number, OD: Right eye, OS: Left eye. R3 OD receiving the commercial HA formulation was used only for clinical evaluations. Data points represent individual measurements on the indicated rabbit.

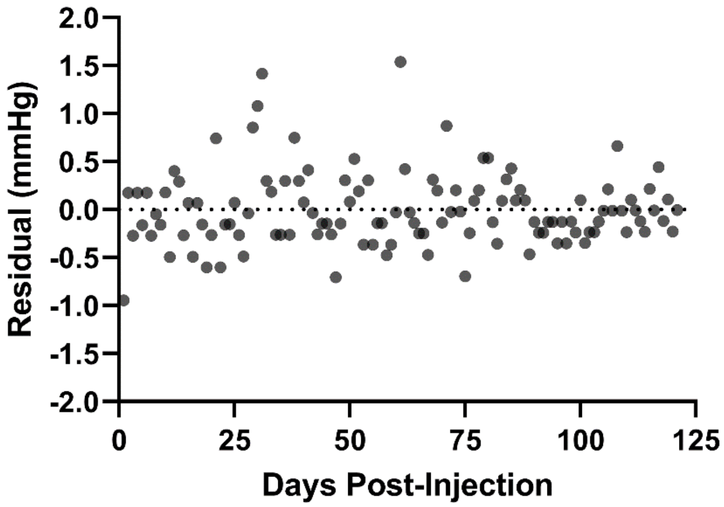

Figure S4: Residual plot for Sham group corresponding to Figure 2B (main text). These residuals were found to fail the test of homoscedasticity ($p < 0.001$) and normality ($p < 0.001$), suggesting a limitation of using linear regression to model these data. However, we elected to accept the linear regression model as it appears to reasonably describe the observed IOP trends. Data are from two eyes. Statistical significant was made at $p < 0.05$.

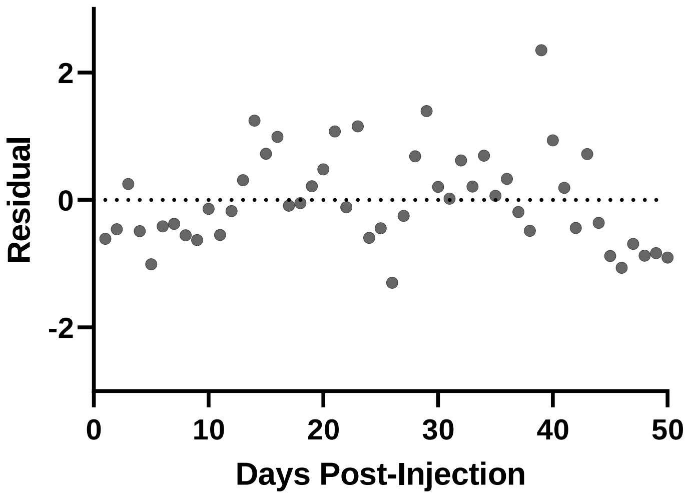

Figure S5: Y-residuals versus time for linear regression of Single HA data. These data passed both the test for normality (p = 0.41) and the test for homoscedasticity (p = 0.12), suggesting evidence is not present to reject the use of the linear regression model. Data are from two eyes. Statistical significant was made at p <0.05.

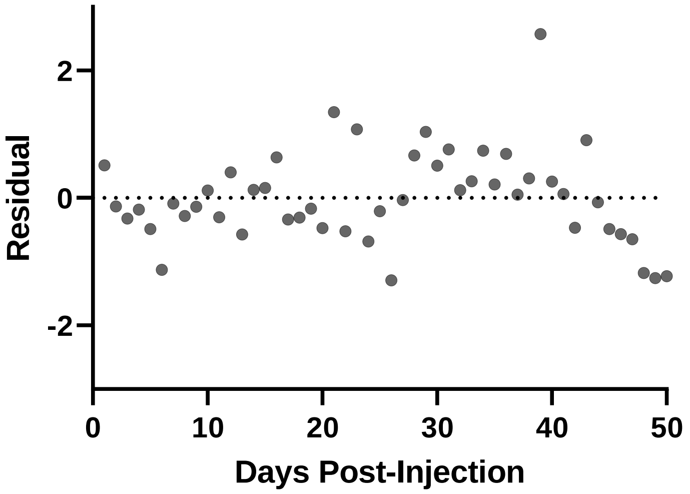

Figure S6: Y-residuals versus time for linear regression of Double HA data. These data passed both the test of normality (p = 0.33) and the test for homoscedasticity (p = 0.37), suggesting evidence is not present to reject the use of the linear regression model. Data are from two eyes. Statistical significant was made at p <0.05.

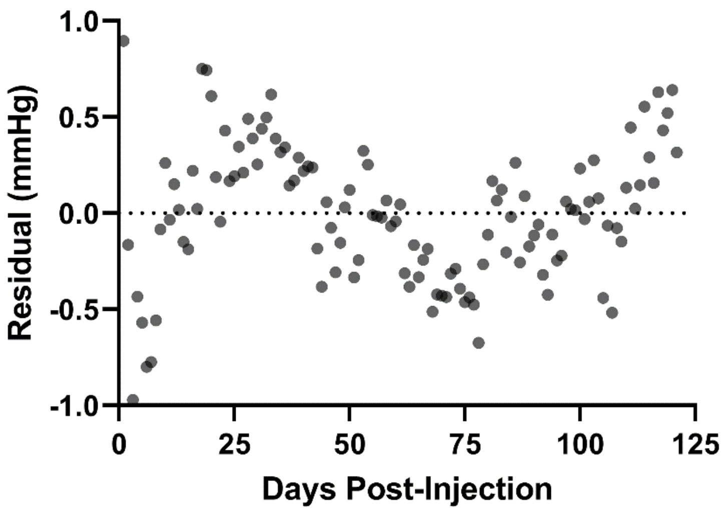

Figure S7. Y-residuals vs. time for the HA-XL group were found to pass the test of normality (p = 0.21) but fail the test of homoscedasticity ($p < 0.0001$), with the latter indicating a limitation of the chosen linear regression model. However, we elected to accept the linear regression model as it appears to reasonably describe the observed IOP trends. Data are from seven eyes. Statistical significant was made at p <0.05.

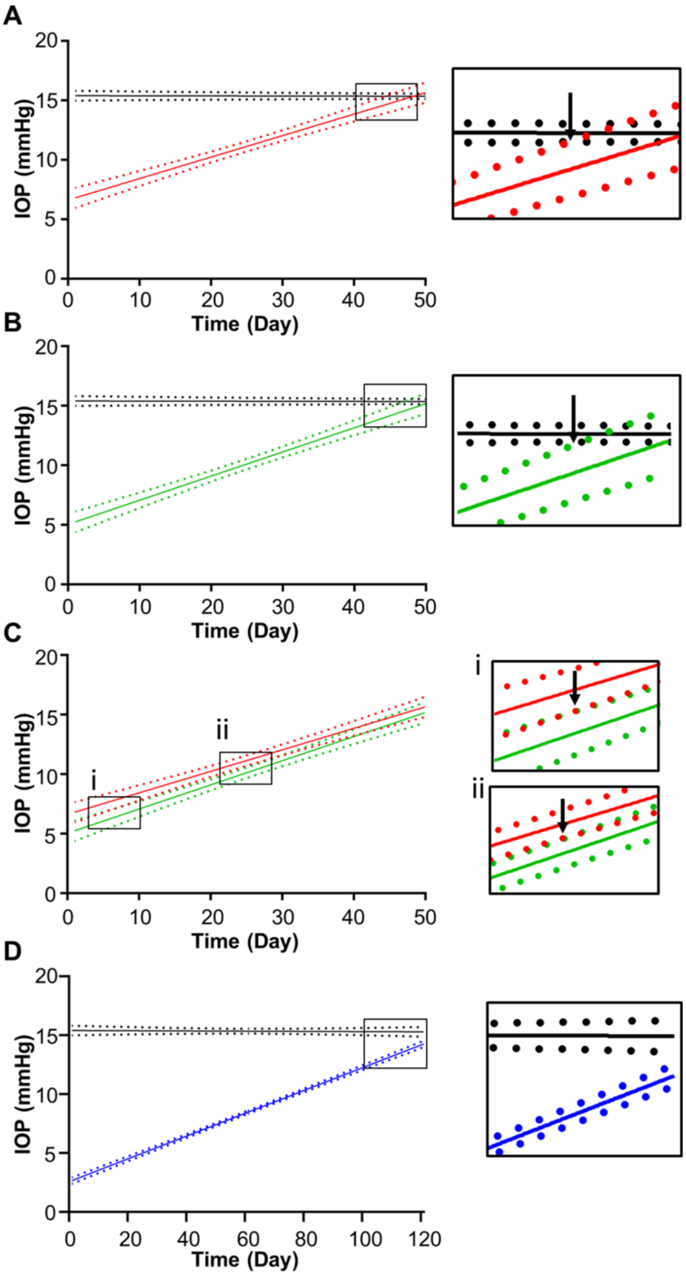

Figure S8. Regression lines (solid lines) with 95% confidence bands (dotted lines) plotted as IOP versus time post-injection. Insets show the intersection (indicated with arrows) of the 95% confidence bands. These bands were not overlapping through A) Day 43 for single HA compared to Sham, B) Day 45 for Double HA compared to Sham. C) For Single HA compared to Double HA, the confidence bands were not overlapping from Day 8 through Day 28. D) Confidence bands of the HA-XL treated eyes and Sham eyes did not overlap throughout the entire duration of the study. Results are from seven eyes (HA-XL group) or two eyes per group (Sham, single HA, and double HA groups).

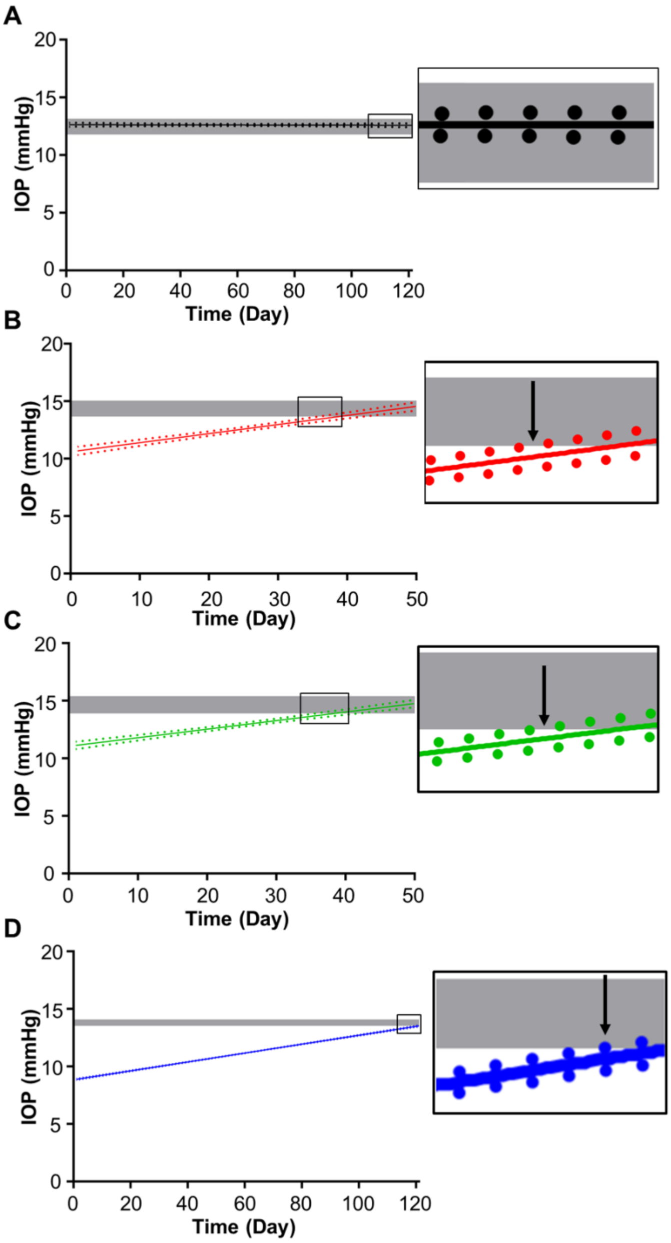

Figure S9. IOP post-injection was analyzed using linear regression and compared to baseline IOP. Regression lines (solid line) with 95% confidence bands (dotted lines) are plotted in addition to 95% confidence intervals (gray shading) for the respective baseline IOP values. Insets show the intersection (indicated with arrows) of the 95% baseline confidence interval with 95% post-injection confidence bands. These intervals/bands A) intersected for all time points in the Sham group, B) did not intersect through Day 35 for Single HA, C) did not intersect through Day 35 for Double HA, and D) did not intersect through Day 119 for the HA-XL group. Results are from seven eyes (HA-XL group) or two eyes per group (Sham, single HA, and double HA groups).

Table S1. Regression parameters for Sham, Single HA, Double HA, and HA-XL treatment group Delta IOP over time, as shown in Figure 2A/2B (main text). The 95% confidence intervals are shown in parentheses.

Treatment Group	Slope [mmHg/Day]	Y-intercept [mmHg]	R-squared
Sham	-0.0003691 (-0.002730 to 0.001992)	0.1621 (-0.003847 to 0.3281)	0.0003949
Single HA	0.07225 (0.06024 to 0.08425)	-3.347 (-3.699 to -2.996)	0.5927
Double HA	0.08109 (0.06888 to 0.09330)	-3.979 (-4.337 to -3.622)	0.6393
HA-XL	0.03867 (0.03711 to 0.04024)	-4.975 (-5.085 to -4.865)	0.7354

Table S2. Regression parameters for Sham, Single HA, Double HA, and HA-XL treatment group IOP regressed over time. The 95% confidence intervals are shown in parentheses.

Treatment Group	Slope [mmHg/Day]	Y-intercept [mmHg]	R-squared
Sham	-0.0003691 (-0.002880 to 0.002142)	12.64 (12.46 to 12.82)	0.0003492
Single HA	0.07909 (0.06631 to 0.09186)	10.60 (10.23 to 10.98)	0.6062
Double HA	0.07426 (0.06284 to 0.08567)	11.05 (10.72 to 11.39)	0.6298
HA-XL	0.03867 (0.03732 to 0.04001)	8.85 (8.76 to 8.95)	0.7901

Table S3. Baseline IOP means with 95% confidence intervals shown in parentheses.

Treatment Group	Mean (mmHg)
Sham	12.48 (11.79 to 13.16)
Single HA	14.36 (13.67 to 15.06)
Double HA	14.65 (13.91 to 15.40)
HA-XL	13.82 (13.54 to 14.09)
